# The hypothetical cost-conflict monitor: is it a possible trigger for conflict-driven control mechanisms in the human brain?

**DOI:** 10.3389/fncom.2014.00077

**Published:** 2014-07-21

**Authors:** Sareh Zendehrouh, Shahriar Gharibzadeh, Farzad Towhidkhah

**Affiliations:** Biomedical Engineering Faculty, Amirkabir University of TechnologyTehran, Iran

**Keywords:** performance monitoring, cognitive control, conflict-driven control, monitor-controller networks, feedback-related negativity

Flexible goal-directed behavior requires a performance monitoring system to monitor behavioral consequences in order to detect the need for further adjustments and control. When a failure in performance is detected by the monitoring system, some signals are transmitted to the brain structures responsible for control implementation. Evidences suggest the anterior cingulate cortex (ACC) (Carter et al., 1998; Gehring and Knight, 2000; MacDonald et al., 2000; Ferdinand et al., 2012) and the lateral prefrontal cortex (lPFC) (MacDonald et al., 2000; Ridderinkhof et al., 2004a,b) as the neural correlates of performance monitoring and control implementation systems, respectively. The interaction of these two systems appears to modulate some components of event-related brain potentials (ERPs) linked with performance monitoring such as the error-related negativity (ERN), the N200, and the feedback-related negativity (FRN) (Gruendler et al., 2011). The ERN is an ERP component that begins close to the time of the erroneous response in speeded response time tasks and peaks about 100 ms later (Gehring et al., 1993). The N200 is another negative deflection in ERP that peaks between 200 and 400 ms after stimulus onset, prior to the response execution, on correct trials of cognitive control experiments (Olvet and Hajcak, 2008). The FRN as one of the most studied components is a negative-going deflection observed 230–330 ms following outcome presentation (Miltner et al., 1997) in gambling and trial-and-error learning tasks (Holroyd et al., 2006). Source localization studies show the neural source of the FRN to be located most probably in the ACC (Miltner et al., 1997; Gehring and Willoughby, 2002; Bellebaum and Daum, 2008; Hauser et al., 2014).

The central question in the interaction of performance monitoring and control systems is how the brain determines the need to recruit the intervention of control structures. The reinforcement learning (RL) account of performance monitoring and control is one of the influential theories to the field (Holroyd and Coles, 2002; Holroyd et al., 2005). The theory is based on the physiological evidences that reveal the similarity of the phasic activity of the mesencephalic dopamine system and reward prediction errors (RPEs) in temporal difference models of learning (Suri, 2002). The theory holds that the monitor is located in the basal ganglia, which produces RPE signals that indicate when events are better or worse than expected. These RPEs are used by the ACC to improve performance on the task at hand (Holroyd et al., 2005). According to the RL model, negative RPEs sent to the ACC generate the ERN and the FRN. Another prominent theory, the conflict-monitoring theory (CMT) proposes that the performance monitoring system monitors for the coactivation of mutually incompatible response tendencies or conflict during response selection. The CMT suggests that the ACC detects response-conflict signal and sends this information to the dorsolateral prefrontal cortex for further adjustment and control (Botvinick et al., 2001; Yeung et al., 2004). Based on this theory, the N2 and the ERN can be described using conflict signal. The CMT argues that the N2 and the ERN are electrophysiologically correlated with pre-response and post-response conflict signals, respectively. However, since no motor response exists after external feedback presentation, the CMT cannot account for the phenomena commencing after feedback onset, e.g., the FRN (Ullsperger et al., 2014). In our previous studies, we have explained the significance of integrating the computational models associated with the RL and the CMT (Zendehrouh et al., 2013, 2014). Since the unification of these two theories depends centrally on conflict signal definition, we propose a hypothetical cost-conflict monitor in the brain that extends the CMT theory to account for post feedback activities in feedback-based learning tasks. Based on this proposal, the FRN can be described using a cost-conflict signal.

The basis for our hypothetical cost-conflict monitor is that: (1) Theoretically, conflict can occur anywhere within the information processing system (Carter and van Veen, 2007). (2) Conflict-driven control is domain-specific suggested to be mediated by multiple, independent, and parallel-operating conflict monitor-controller loops in the brain (Egner, 2008). (3) The appraisal of costs and benefits associated with different candidate actions is a key aspect of decision-making.

The Delay-based and the effort-based costs (effort needed to perform an action in order to obtain a reward) are two types of costs that bias decision making (Floresco et al., 2008). In delay-based tasks, as the time passes, the subjective value of a reward is discounted hyperbolically (Green and Myerson, 2004). Also, the aversiveness of a negative event decreases hyperbolically with time (Murphy et al., 2001). Evidences suggest that discounting can happen across many reward types, reward magnitudes, and several timescales even in the order of tens of milliseconds (Haith et al., 2012). In this paper, it is hypothesized that in feedback-based learning tasks, the participants are faced with delay-based evaluations. Therefore, in these tasks, the time interval between response selection and feedback presentation gives rise to a cost. This delay elevates the cost of the rewarded outcome and reduces the cost of the non-rewarded outcome associated with the selected action. In fact, the conflict can be produced by simultaneous activation of the expected costs of possible outcomes that are mutually exclusive. Therefore, when a cost-conflict is detected by the monitoring system, the regulatory mechanism implements the required control, e.g., by modifying the excitatory weights to the response units. The cost-conflict signal that may occur between expected costs can show the amount of subjective transient uncertainty about what will happen that increases with time (delay) until receiving the actual outcome. The cost-conflict signal can also be viewed in the context of the emerging field of neuroeconomics as an ambiguity signal that may be present during decision-making. Ambiguity is defined as a lack of confidence in probability assignment to the possible outcomes (Kishida et al., 2010). This is consistent with investigations suggesting the existence of an ambiguity-sensitive mechanism in the ventromedial prefrontal cortex (vmPFC) (Glimcher and Rustichini, 2004), and also with the role of this area in delay cost coding (Prévost et al., 2010; Rushworth et al., 2011; Dreher, 2013).

This proposal can be validated by performing simple gambling games or probabilistic reinforcement learning tasks with feedback-timing manipulations at the timescale of milliseconds while measuring the brain responses with functional magnetic resonance imaging (fMRI) and electroencephalography (EEG) to identify the contributions of the ACC and the vmPFC in those tasks. Especially, the behaviors of addicted and depressed individuals in these tasks that show anomalies in value based decision making (Sharp et al., 2012) can be beneficial.

Therefore, the cost-conflict monitor as an independent and parallel loop to the response-conflict monitor detects the conflict between the costs of likely outcomes of the selected action and uses this information to adjust the behavior for the future, thereby implements trial-by-trial adjustments. Surely, this proposal is speculative and further experimental studies and research is needed to evaluate its merit. However, the proposal can provide promising avenues toward the unification of computational models associated with the RL and the CMT.

## Conflict of interest statement

The authors declare that the research was conducted in the absence of any commercial or financial relationships that could be construed as a potential conflict of interest.
